# Effect of Ultrasonic Vibration on Mechanical Properties of 3D Printing Non-Crystalline and Semi-Crystalline Polymers

**DOI:** 10.3390/ma11050826

**Published:** 2018-05-17

**Authors:** Guiwei Li, Ji Zhao, Wenzheng Wu, Jili Jiang, Bofan Wang, Hao Jiang, Jerry Ying Hsi Fuh

**Affiliations:** 1 School of Mechanical Science and Engineering, Jilin University, Renmin Street 5988, Changchun 130025, China; ligw15@mails.jlu.edu.cn (G.L.); jzhao@jlu.edu.cn (J.Z.); jianghao17@mails.jlu.edu.cn (J.J.); wangbf1415@mails.jlu.edu.cn (B.W.); jianghao17@mails.jlu.edu.cn (H.J.); 2 Department of Mechanical Engineering, National University of Singapore, Singapore 117576, Singapore; jerry.fuh@nus.edu.sg

**Keywords:** 3D printing, FDM, additive manufacturing, ultrasonic strengthening, mechanical properties, polymer

## Abstract

Fused deposition modeling 3D printing has become the most widely used additive manufacturing technology because of its low manufacturing cost and simple manufacturing process. However, the mechanical properties of the 3D printing parts are not satisfactory. Certain pressure and ultrasonic vibration were applied to 3D printed samples to study the effect on the mechanical properties of 3D printed non-crystalline and semi-crystalline polymers. The tensile strength of the semi-crystalline polymer polylactic acid was increased by 22.83% and the bending strength was increased by 49.05%, which were almost twice the percentage increase in the tensile strength and five times the percentage increase in the bending strength of the non-crystalline polymer acrylonitrile butadiene styrene with ultrasonic strengthening. The dynamic mechanical properties of the non-crystalline and semi-crystalline polymers were both improved after ultrasonic enhancement. Employing ultrasonic energy can significantly improve the mechanical properties of samples without modifying the 3D printed material or adjusting the forming process parameters.

## 1. Introduction

In fused deposition modeling (FDM) 3D printing technology, the printed material is heated by nozzle, melts, and is ejected with a certain pressure through the nozzle. At the same time, the nozzle moves along the XY plane, and the extruded material is fused with the former layer. After a deposited layer is completed, the work table vertically descends the thickness of one layer, and the melting and deposition is continued until the entire part is completed [1,2]. Unlike traditional manufacturing technology, FDM 3D printing allows a customized sample with a complex structure to be rapidly formed using thermoplastic materials [3,4,5]. Thermoplastic materials such as acrylonitrile butadiene styrene (ABS) and polylactic acid (PLA) are the most widely used FDM 3D printed materials [6,7]. 3D printed ABS parts exhibit good impact resistance, stable product size, good chemical resistance [8], and excellent electrical properties [9] and are widely used in machinery, automobiles, electronic and electrical appliances, and instrumentation [10,11,12]. 3D printed PLA parts are biodegradable [13], exhibit good biocompatibility and antibacterial properties and are widely used in biomedical, educational, and textile industries [14,15,16,17]. However, because of the raster-by-raster melting and accumulating formation characteristics of FDM technology, several pores are formed in the sample, and the deposited rasters are not completely integrated into one [18,19,20,21]. The mechanical properties of the formed sample are thus poorer than those of injection molded samples [22], which restricts the development and application of FDM 3D printing technology.

Many researchers have aimed to improve the mechanical properties of FDM parts. Through the development of iron/ABS and copper/ABS composite materials, Nikzad et al. [23] observed that the addition of metal can produce printed samples with improved stiffness and flexibility. Lederle et al. [24] observed that, under a nitrogen atmosphere, the oxidative decomposition of ABS materials is inhibited during the printing process, thereby improving the mechanical properties of 3D printed ABS samples. Li et al. [25] performed comparative analysis of the tensile strength, bending strength, and dynamic mechanical properties of 3D printed PLA samples and PLA composites enhanced by continuous carbon fibers. Jin et al. [26] used dichloromethane steam to dissolve the staircase on the surface of PLA samples and studied the effect of a chemical finishing process on the tensile mechanical properties of 3D printed samples. Adel et al. [27] developed a polishing technique to melts the staircase on the surface of FDM products by hot air jet. The effects of carbon and glass fibers on the mechanical properties and fracture modes of 3D printed thermoplastic samples were analyzed by Goh et al. [28]. Dawoud et al. [29] studied the effect of varying the raster angle and gap dimensions on the tensile and bending mechanical properties of 3D printed samples.

The above studies focused on improving the mechanical properties of FDM 3D printed samples by modifying the 3D printed material or adjusting the forming process parameters. 3D printed ABS samples strengthened by ultrasonic vibrations were studied by a controlled variate method, and the effects of ultrasonic strengthening pressure and ultrasonic strengthening time on the mechanical properties of samples were studied [30,31]. The existing research illustrated that the mechanical properties of 3D printed ABS samples could be improved by ultrasonic vibrations and pointed out the effects of different ultrasonic parameters on the mechanical properties. However, the effects of ultrasonic vibrations on the interface of the printed rasters and the different kinds of polymers still need to be analyzed. For a specified 3D printed material, the mechanical performance of the final printed sample mainly depends on the interdiffusion and re-entanglement between the deposition rasters of the fused polymer inside the sample [32,33]. In this study, the effects of ultrasonic intensification on the mechanical properties of 3D printed non-crystalline and semi-crystalline polymers were compared and analyzed by the idea of entanglement formation across the interface.

## 2. Materials and Methods

### 2.1. Ultrasonic Strengthening Process

**Figure 1:**
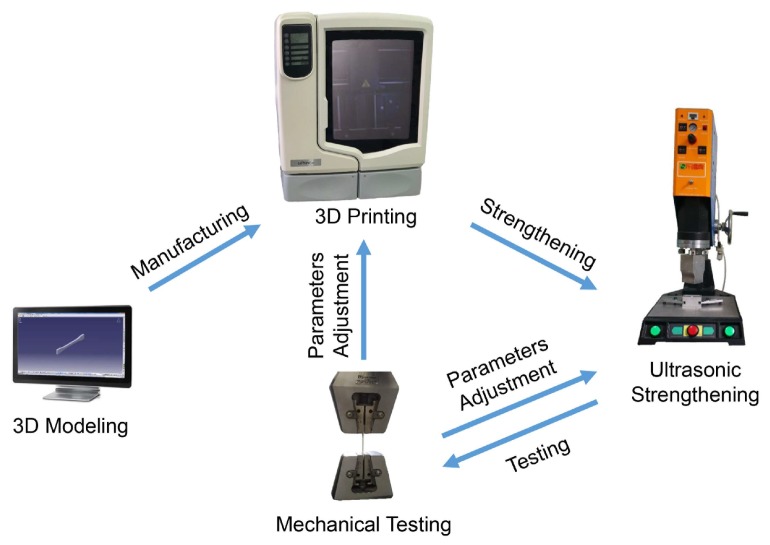
Schematic diagram of ultrasonic strengthening process.

The process used for the ultrasonic enhancement of the 3D printed samples is illustrated in Figure 1. According to the sample size, a 3D model of the sample was built, and the 3D solid sample was formed using the 3D printer in accordance with the section profile information of the sample. The sample was then processed by ultrasonic treatment. The tensile, bending and dynamic mechanical properties of the sample were evaluated, and the printing and ultrasonic intensification parameters were adjusted in an iterative manner.

### 2.2. 3D printing of Mechanical Samples

The 3D printed tensile and bending samples were designed according to ISO 527-2:2012 [34] and ISO 178:2001 [35] standards, respectively. CATIA V5 software was used for the 3D modeling, and a uPrint SE 3D Printer (Stratasys Inc., Eden Prairie, MN, USA) was used to form the non-crystalline polymer samples. The forming material was ABS plusTM-P430 material (Stratasys Inc., Eden Prairie, MN, USA), the printing speed was 15 mm/s, the thickness of the printed layer was 0.254 mm, the printing direction was Y-direction, the raster angle was 45° and the interior of the forming sample was fully filled. The semi-crystalline polymer PLA samples were printed employing an Einstart-S Desktop 3D Printer (Shining 3D Tech Co., Ltd., Hangzhou, China). The forming material was PLA 3D filament (Shining 3D Tech Co., Ltd. Hangzhou, China), the thickness of the printed layer was 0.25 mm, the printing speed was 60 mm/s, and the interior of the forming sample was fully filled.

### 2.3. Ultrasonic Strengthening Experiment

A customized ultrasonic vibration system was employed to strengthen the 3D printed samples. We studied the effects of the main ultrasonic strengthening parameters on the tensile mechanical properties of 3D printed ABS samples by a controlled variate method [30]. The power and frequency were constant. The delay time was the time between the start of the ultrasonic strengthening system and the ultrasonic emission, and the curing time was the time when the horn continued to press on the samples after the ultrasonic emission. They had slight effects on the mechanical properties of the strengthened samples. Weld time was the time that the ultrasonic wave acted on the samples. Pressure and weld time were the main parameters which effected the properties of the strengthened samples. The printed ABS and PLA samples were strengthened using the optimal ultrasonic strengthening parameters. The experimental parameters are listed in Table 1.

For the ultrasonic strengthening process, the FDM 3D printed sample was fixed on the work plate of the ultrasonic strengthening system. The ultrasonic strengthening system was started after the ultrasonic strengthening parameters set. The ultrasonic intensification horn was then contacted with the sample, the horn exerted pressure on the sample, and the ultrasonic vibration was applied. After ultrasonic strengthening, the horn continued to compact the sample for a certain period to prevent warpage of sample. Finally, the horn was retracted to complete the ultrasonic strengthening process.

### 2.4. Mechanical Testing of Ultrasonically Enhanced 3D Printed Samples

To evaluate the effect of the ultrasonic vibration on the mechanical properties of the 3D printed non-crystalline ABS and semi-crystalline PLA polymers, the tensile and bending properties of the 3D printed samples were tested using an INSTRON 5982 electronic universal testing machine (INSTRON, Norwood, MA, USA). according to the ISO 527-2:2012 and ISO 178:2001 standards. The dimensions and loading modes of the test samples are shown in Figures of stress–strain curves. Five mechanical samples were tested for each group of mechanical experiments, the average value was recorded, the standard deviation was marked on the histograms and the sample test result which was the closest to the average value was selected for the stress-strain curve. Scanning electron microscopy (SEM) XL-30 (FEI, Hillsboro, USA) was used to examine the features of the fractured samples and the changes in the interface morphology of the internal printed rasters after ultrasonic intensification. To analyze the effect of ultrasonic vibration on the dynamic mechanical properties of the 3D printed samples, the dynamic mechanical properties of bending samples were tested using a DMA + 450 dynamic mechanical analyzer (MetraviB, Limonest, France). Effects of ultrasonic vibration on the chemical properties of 3D printed PLA samples were analyzed by X-ray diffraction (XRD) D/Max 2500pc (Rigaku, Tokyo, Japan) and differential scanning calorimeter (DSC) Q20 (TA, New Castle, USA).

## 3. Results and Discussion

### 3.1. Effect of Ultrasonic Vibration on Tensile Properties

**Figure 2:**
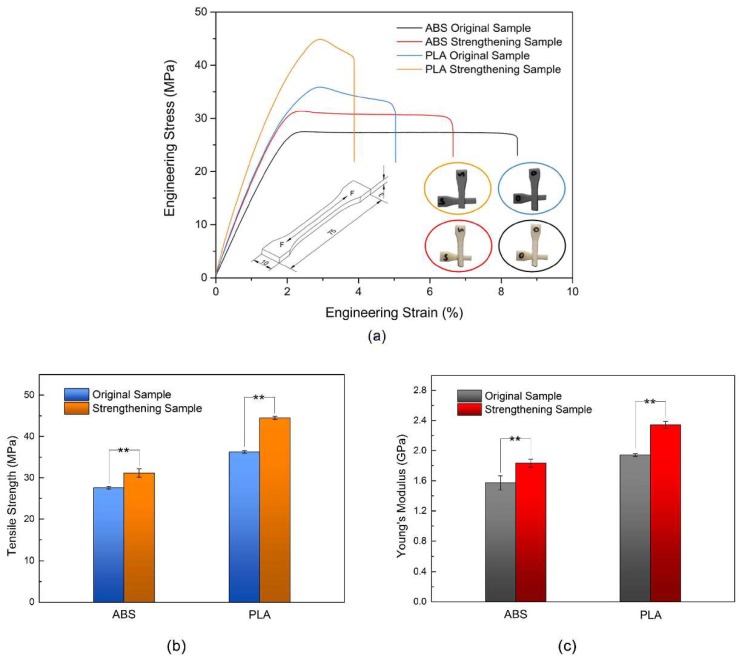
Tensile stress–strain curves (**a**); tensile strength (**b**); and Young’s Modulus (**c**) of original and strengthening samples. Data presented as mean ± standard deviation, ** = *p* < 0.01.

Figure 2a presents stress–strain curves of the tensile tested 3D printed samples. The tensile strength, breaking stress, and tensile modulus of the 3D printed non-crystalline ABS samples and semi-crystalline PLA samples increased, and the elongation at break decreased after the application of ultrasonic vibration to the 3D printed samples. These findings indicate that the maximum tensile load of a 3D printed sample can be increased by ultrasonic enhancement and that the unit elastic deformation can bear a greater load. For the non-crystalline ABS sample, upon reaching the tensile strength of the specimen, the sample entered a stable plastic deformation stage. With increasing strain, the stress remained almost unchanged, and the specimen then directly fractured. For the semi-crystalline PLA samples, upon reaching the tensile strength, the strain slowly decreased until the specimen rapidly fractured. After ultrasonic strengthening, upon reaching the tensile strength of the PLA samples, with increasing strain, the stress rapidly decreased until the sample fractured. However, the tensile stress remained greater than that of the original sample. As shown in the tensile stress–strain curves, the elongations at break of original and ultrasonic-strengthened ABS samples were almost twice of that of PLA samples, respectively. The ABS samples had more cracks on their surfaces and the crazes widened which were mainly due to the toughness of polymer fibers with high orientation in ABS sample was larger than that in PLA sample during plastic deformation. This explained the greater elongation at break of the ABS samples compared with that of the PLA samples.

Figure 2b,c summarizes the tensile test results. The tensile strength and tensile modulus of the original ABS and PLA samples were 27.59 ± 0.32 MPa, 36.22 ± 0.30 MPa, 1.57 ± 0.10 GPa and 1.94 ± 0.02 GPa, respectively. The tensile strength and tensile modulus of the ultrasonic-strengthened ABS and PLA samples were 31.16 ± 1.03 MPa, 44.49 ± 0.38 MPa, 1.83 ± 0.05 GPa and 2.34 ± 0.05 GPa, respectively. A post hoc Tukey’s test revealed that tensile properties of the two types of ultrasonic-strengthened samples were significantly different from that of the original samples (*p* < 0.01). After ultrasonic enhancement, the tensile strength and Young’s modulus of the 3D printed ABS sample increased by 12.94% and 16.56%, respectively, and the tensile strength and Young’s modulus of the PLA sample increased by 22.83% and 20.62%, respectively. The improvement of the tensile mechanical properties of the 3D printed PLA sample was thus greater than that of the ABS sample, which may be related to the 3D printers used. The ABS sample was formed using a high-precision desktop 3D printer with an air-tight forming chamber. The quality of the forming sample was better, and the increase in the tensile mechanical properties was smaller after ultrasonic strengthening. In contrast, the PLA sample was printed using an ordinary low-budget desktop 3D printer.

**Figure 3:**
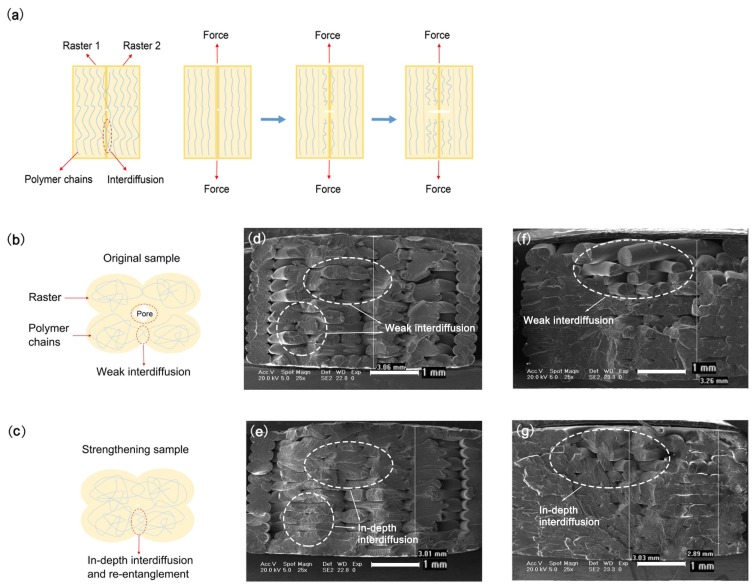
Schematic illustration of tensile breakage of: 3D printed samples (**a**); microcosmic interface of original (**b**); and ultrasonic-strengthened (**c**) samples. SEM images of fractured surfaces: original ABS samples (**d**); ultrasonic-strengthened ABS samples (**e**); original PLA samples (**f**); and ultrasonic-strengthened PLA samples (**g**).

Under the application of tensile stress, the irregular coil chain inside a polymer unfolds or orientates along the direction of stress. The molecular chain that is in the high-stretching state will break firstly under the action of stress, which will cause a stress concentration inside the material, accelerate the fracture of the molecular chain, and gradually cause the formation of micro cracks. With crack growth, the entire sample will finally fracture. The tensile strength of a 3D printed sample is determined by the interdiffusion and re-entanglement of the polymer melt through raster interfaces in the process of raster-by-raster accumulating forming in addition to the internal chain of the sample material. As illustrated in Figure 3a, the molecular chain inside the 3D printed sample mainly spreads or orientates along the direction of the printed rasters. When a tensile stress is applied, the molecular chain at the interface of the printed raster will firstly break in a high-stress state because of the effect of micro holes and then produce cracks. The cracks will expand until the entire sample fractures.

Figure 3b,c shows the effect of ultrasonic vibration on the interface. Figure 3d,e presents SEM images of the fracture surface of the original and ultrasonic-strengthened ABS samples. Figure 3f,g presents SEM images of the fracture surface of the original and ultrasonic-strengthened PLA samples. Before ultrasonic strengthening, some larger gaps appeared inside the 3D printed sample, the interface of rasters inside the sample was weaker, and the outline of a single raster could be clearly distinguished. Especially for the PLA sample, there was almost no mutual penetration between the rasters of the surface layers. After ultrasonic strengthening, the spaces within both samples were reduced and even disappeared. In some sections, the rasters almost melted together, making it difficult to distinguish the outlines of single rasters. Rasters of the surface layers that did not melt together were also deeply penetrated and fused with each other. This finding indicates that ultrasonic strengthening can make the polymer diffuse and re-entangle deeply at the interface between rasters and increase the interfacial bonding area and bonding strength between printed rasters. After ultrasonic strengthening, the polymer molecular chain at the interface was deeper interdiffused and re-entangled, which reduced the number of pores at the interface and resulted in a more uniform distribution of the internal stress when the sample was subjected to tensile loading. As shown in Figure 3d–g, the thicknesses of ABS strengthened samples were slightly reduced, while the thicknesses of PLA strengthened samples were reduced by almost 10%. The other geometric dimensions were almost unchanged. Therefore, the tensile mechanical properties of the samples were enhanced mainly due to the deeper interdiffused and re-entangled. This can also illustrate why the improvement of the tensile mechanical properties of PLA sample was greater.

### 3.2. Effect of Ultrasonic Vibration on Bending Properties

**Figure 4:**
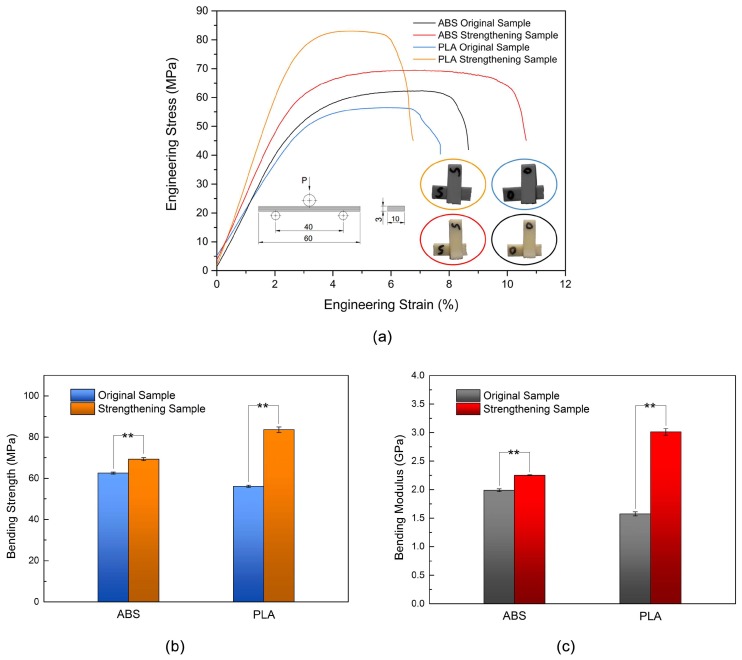
Bending stress–strain curves (**a**); bending strength (**b**); and bending modulus (**c**) of original and strengthening samples. Data presented as mean ± standard deviation, ** = *p* < 0.01.

Figure 4a presents the stress–strain curves of the three-point bending test samples. The bending strength, fracture stress, and bending modulus of the 3D printed ABS and PLA samples increased after ultrasonic strengthening, and the ABS sample had a greater bending fracture strain than the PLA sample. This finding indicates that the maximum bending load on the sample can be increased with ultrasonic strengthening and that the unit bending deformation can bear a greater bending load. For the ABS sample, the sample entered a stable plastic deformation phase when the stress approached the bending strength. With increasing strain, the stress was almost constant, and then the sample rapidly fractured. The bending strength and bending toughness of the ABS samples significantly increased with ultrasonic strengthening. The bending fracture strain of the PLA sample was lower than that of the ABS sample. The fracture strain of the PLA sample was reduced after ultrasonic strengthening; however, the bending strength and bending modulus of the sample significantly increased, and the bending toughness of the sample also improved.

Figure 4b,c summarizes the bending test results. The bending strength and bending modulus of the original ABS and PLA samples were 62.50 ± 0.47 MPa, 56.06 ± 0.49 MPa, 1.99 ± 0.02 GPa and 1.57 ± 0.04 GPa, respectively. The bending strength and bending modulus of the ultrasonic-strengthened ABS and PLA samples were 69.26 ± 0.72 MPa, 83.56 ± 1.40 MPa, 2.25 ± 0.01 GPa and 3.01 ± 0.06 GPa, respectively. The bending properties of the two types of ultrasonic-strengthened samples were significantly different from that of the original samples revealed by the Tukey’s test (*p* < 0.01). The bending strength and bending modulus of the 3D printed PLA increased by 49.05% and 91.72%, respectively, which were almost five times the percentage increase in the bending strength and seven times the percentage increase in the bending modulus of the 3D printed ABS with ultrasonic strengthening. The improvement of the bending mechanical properties of the 3D printed PLA with ultrasonic strengthening was much greater than that of the ABS samples, which may be related to the forming quality of the 3D printers used. The rasters in the ABS sample were bonded evenly from bottom to top. In the PLA sample, the quality of the surface layers was poor. When the specimen was subjected to bending load and deformation, the printed rasters of the surface layers could hardly bear load. After ultrasonic strengthening, the rasters of the surface layers also fused with each other. The entire specimen could bear bending load, and the improvement of the bending mechanical properties of sample was much more significant.

**Figure 5:**
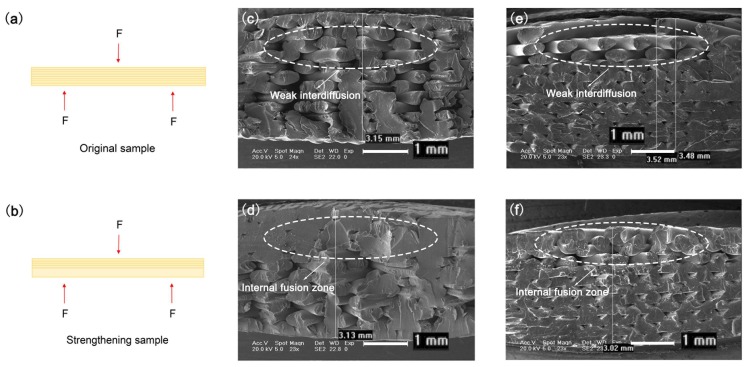
Schematic illustration showing: bending load of original samples (**a**); and ultrasonic-strengthened samples (**b**). SEM images of fractured surfaces of bending samples: original ABS samples (**c**); ultrasonic-strengthened ABS samples (**d**); original PLA samples (**e**); and ultrasonic-strengthened PLA samples (**f**).

Figure 5 presents cross-sectional SEM images of the bending fracture samples. The bending load of the sample is shown in Figure 5a,b. After the ultrasonic strengthening, several sedimentary layers were fused into a single deposited layer in the internal fusion zone of the sample. The bending load required for unit bending strain increased; thus, the bending mechanical properties were improved. Figure 5c,d presents SEM images showing the fractured surface of the bending ABS samples without and with ultrasonic strengthening, respectively. Figure 5e,f presents SEM images of the fracture surface of the original and ultrasonic-strengthened PLA samples. The fractured surfaces are similar to that of the tensile samples shown in Figure 3b,c. Before ultrasonic enhancement, some large gaps appeared within the 3D printed parts, and the outline of a single raster could be clearly distinguished. Especially in the PLA sample, there was almost no penetration or fusion between the rasters of the surface layers. After ultrasonic enhancement, the interior of the ABS sample was almost fused together. With deeper interdiffusion and re-entanglement between the rasters and only a few gaps, it is difficult to distinguish the outlines of the rasters. After ultrasonic strengthening, the gap of the PLA sample was significantly reduced, and the interfacial area between rasters increased. In the part near the surface layer, the rasters were also fused together, whereas, in the original sample, they were seldom bonded. Therefore, the bending properties of the samples were greatly improved with ultrasonic strengthening. In addition, the shrinkage of PLA samples caused by the fusion between rasters after ultrasonic strengthening was bigger than that of ABS samples. For these reasons, the bending mechanical properties of the 3D printed PLA with ultrasonic strengthening were much greater than that of the ABS samples.

### 3.3. Effect of Ultrasonic Vibration on Dynamic Mechanical Properties

**Figure 6:**
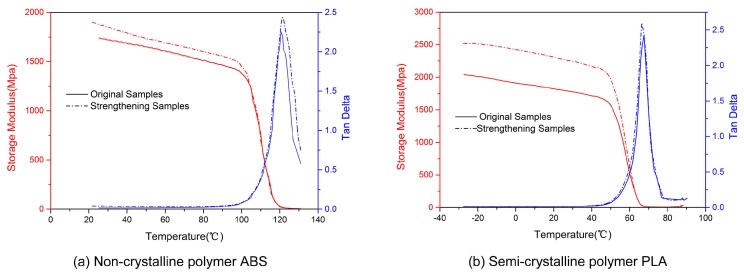
Storage modulus and loss tangent as a function of temperature for the original and ultrasonic-strengthened samples: non-crystalline polymer ABS (**a**); and semi-crystalline polymer PLA (**b**).

Curves of the storage modulus and loss tangent as a function of temperature of the original and ultrasonic-strengthened ABS and PLA samples are presented in Figure 6. The maximum storage modulus values of the two types of ultrasonic-strengthened samples were greater than the original values throughout the storage modulus curves. The storage modulus of the 3D printed PLA samples was increased by almost 25% at below 45 °C, which was almost five times the percentage increase in that of the 3D printed ABS samples at below 90 °C with ultrasonic strengthening. Existing studies showed that the molecular motion at the internal interface of the sample was limited when the interaction between the molecular chains of the interface was enhanced, thereby enhancing the stiffness of the sample and increasing the storage modulus [23,36]. As shown in Figure 3 and Figure 5, SEM observation of the sample sections revealed that the densities of the ultrasonic-strengthened samples were greater than that of the original samples. Therefore, the internal pores of the sample may have decreased in size, and the rasters and layers may have grown closer with an increase in bonding strength when the ultrasonic intensification was employed. The polymer molecular chain at the interface was deeper interdiffused and re-entangled. Thus, the storage modulus was increased.

The peak values of loss tangents of the two materials were both increased by almost 8%. The sample is more dissipative as tan δ increases [37]. The increased loss tangent may be due to the deeper interdiffused of the polymer chain at the interface, which increased the friction in the molecular layer. This means that the strengthened samples are more suitable for shock-absorbing or soundproofing. The temperature at where the peak of tan δ occurs represents glass transition temperature of the samples [35]. The peak temperatures of the two materials showed no obvious change after ultrasonic strengthening, which meant the glass transition ability of the two materials did not change.

### 3.4. Effect of Ultrasonic Vibration on the Chemical Properties 

**Figure 7:**
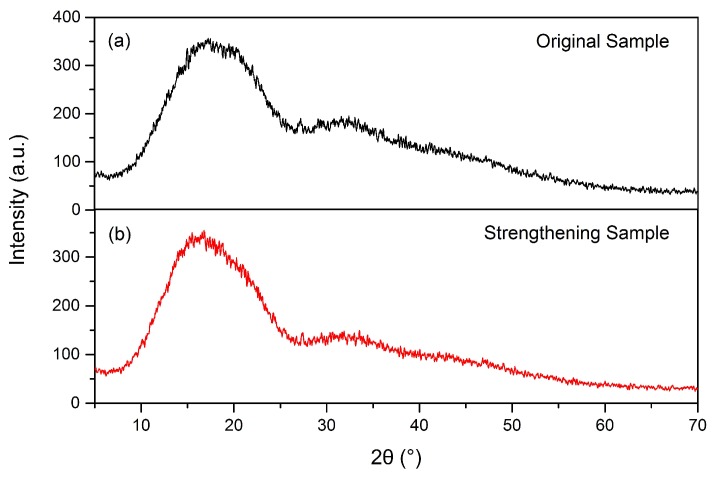
XRD patterns of the original (**a**) and ultrasonic-strengthened (**b**) PLA samples.

Figure 7a,b presents XRD patterns of the ultrasonic acting surface of the original and ultrasonic-strengthened PLA samples. They both show only a broad halo diffraction peak at almost the same position. No new crystal phases were found in 3D printed PLA sample after ultrasonic strengthening. This is because, when the ultrasonic vibration is applied to the printed parts, the friction between the molecules at the interface between rasters resulted in deformation and an increase of temperature for a short period [38]. The ultrasonic strengthening time was only approximately one second, which was too short to crystallize for the semi-crystalline polymer PLA.

**Figure 8:**
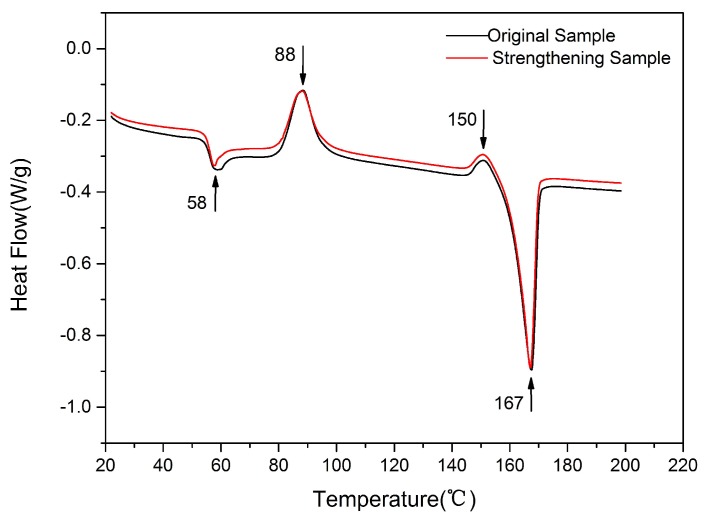
DSC patterns of the original and ultrasonic-strengthened PLA samples.

Figure 8 shows the DSC curves of the original and the ultrasonic-strengthened 3D printed PLA samples. The endothermic peaks and exothermic peaks of the samples were basically coincident throughout the curves. The further endothermic heat flow which represented glass transition occurred at around 58 °C. The melting peaks at around 167 °C corresponded to melting of the 3D printed materials. The low-temperature exothermic peaks that occurred at around 88 °C were mainly caused by incomplete crystallization. The high-temperature exothermic peaks occurred at around 150 °C were caused by the melting recrystallization of a small number of unstableαcrystals to the more stable crystals during the heating process [39,40]. The crystallinity of the samples was calculated by [(Δ*H*_m_ − Δ*H*_c_)/Δ*H*_f_] × 100%, where Δ*H*_m_ is melting enthalpy, Δ*H*_c_ is the enthalpy of crystallization on heating, and Δ*H*_f_ is the enthalpy of melting for a fully crystalline PLA with a value of 93 J/g [41]. The crystallinities of the original sample and the ultrasonic-strengthened sample were about 1.60% and 1.67%, respectively. The low crystallinities inside the samples were mainly due to the high cooling rate during the printing process [42]. Although the crystallinity of 3D printed semi-crystalline polymer PLA was low and the sample was almost in an unstable amorphous state, the glass transition temperature, melting point and crystallinity of the sample changed little after the rapid input of ultrasonic vibration. According to XRD analysis, there was no new crystallite precipitation after ultrasonic strengthening. Therefore, ultrasonic vibration did not change the chemical properties of the printed materials, but only improved the mechanical properties of the samples from the manufacturing process.

## 4. Conclusions

In this study, the effect of ultrasonic vibration on the mechanical performance of 3D printed non-crystalline and semi-crystalline polymers was analyzed. The results indicated that, with ultrasonic strengthening, the tensile, bending, and dynamic mechanical properties of the non-crystalline polymer ABS and semi-crystalline polymer PLA were greatly improved. In addition, the ultrasonic vibration did not affect the properties of the forming material itself. The improvement of the mechanical properties with ultrasonic strengthening was mainly attributed to the deeper interdiffusion and re-entanglement at the raster interface caused by the ultrasonic vibration. The gaps were reduced and even removed, the interfacial area was increased, and the bonding strength between the rasters was improved. The ultrasonic enhancement compensated for the pores in the forming samples and defects in the printed rasters caused by the raster-by-raster and layer-by-layer characteristics of FDM technology. The mechanical properties of FDM 3D printed parts were improved without modifying the 3D printed material or adjusting the forming process parameters. However, the types of parts that could practically be improved through sonication are still only solid parts. This process still needs to be improved to strengthen the gel parts and hollow parts. The ultrasonic-strengthened area is limited to the horn area and the ultrasonic-strengthened depth is determined by the input ultrasonic vibrational energy. The ultrasonic strengthening system may be combined with FDM 3D printer into one device. The sample can be strengthened by printing several layers at one time during the process of 3D printing. It will expand the application of low-cost FDM 3D printed parts in industrial and other fields.

## Figures and Tables

**Table 1:** Ultrasonic strengthening parameters.

Factor	Value	Unit
Power	2	kw
Frequency	20	kHz
Pressure	3.5	kg/cm^2^
Delay time	0.49	s
Weld time	0.65	s
Curing time	0.50	s
